# Supplementary material for: Polluted white dwarfs reveal exotic mantle rock types on exoplanets in our solar neighborhood
Source: Nat Commun. 2021 Nov 2;12:6168. doi: 10.1038/s41467-021-26403-8 (PMC8563750; doi:10.1038/s41467-021-26403-8)
Supplement: Supplementary file 1 — Supplementary Information [file 41467_2021_26403_MOESM1_ESM.pdf]

Supplementary Data & Equations

Polluted white dwarf compositions are typically reported as either log(Z/Y) = *log[n(Z)/n(H)]* or log(Z/Y) = *log[n(Z)/n(He)]*, where *n(Z)* is the number of atoms of the element of interest, Z, and in the denominator, *n(Y)* is the number of atoms of either H or He, depending upon which dominates a PWD's atmosphere. Since Y is the same for any given PWD, cation fractions for any given star can be obtained by renormalization irrespective of Y, so that $10^{[Mg/Y]} + 10^{[Si/Y]} + 10^{[Ca/Y]} + 10^{[Fe/Y]}$ = 100% = Mg + Si + Ca + Fe, on the basis of numbers of atoms; multiplication by atomic weights and renormalization provides weight % values. In the Hypatia Catalog, main sequence star compositions are reported as a difference in log concentrations relative to the Sun (always as [Z/H]) and thus require a correction using solar elemental abundances (Lodders et al. 2009). Cations of meteorites are often plotted as Z/Si, but reported values are given as cation or oxide percentages. Error bars for PWDs use reported uncertainties in the literature to derive the standard deviations from 1,000 Monte Carlo simulations (assuming a normal distribution) to arrive at an average uncertainty; median reported uncertainties in the literature (which in units of log(Z/Y) are: ± 0.18 dex Mg; ± 0.15 dex Si; ± 0.156 dex Ca; ± 0.12 dex Fe). Error bars on Hypatia compositions are propagated from the compositional "spread" of Hinkel et al. (2014, 2016); Monte Carlo simulations on analytical uncertainties reported in Hinkel et al. (2014) would yield very much smaller error bars than reported here, but it is not clear that such uncertainties are fully representative of star composition uncertainties (see Hinkel et al. 2014). Uncertainties for Earth and Martian compositions, where shown, represent standard deviations from McDonough and Sun (1995) and Yoshizaki and McDonough (2020), and "high" and "low" values of Khan and Connelly (2008).

Calculating Bulk Silicate Planet Compositions

In addition to comparing bulk materials, we also calculate Bulk Silicate Planet (BSP) compositions for PWDs, using the methods applied to Hypatia stars in Putirka and Rarick (2019) (Table A2). We compare these to silicate compositions from our Solar System. Oxygen is sufficiently abundant in parent FGMK stars (Unterborn and Panero 2017; Putirka and Rarick 2019) and PWDs (Doyle et al. 2019) so that Mg, Si and Ca are almost certainly fully oxidized (to MgO, SiO_2 and CaO) and some fraction of Fe is oxidized to FeO, depending upon a planet's oxygen fugacity (fO_2). As a proxy for fO_2, we apply the approach of Putirka and Rarick (2019) using: $\alpha_{Fe} = Fe^{BSP}/Fe^{BP}$, where Fe^{BSP} is the cation fraction of Fe in the bulk silicate planet (crust + mantle), and Fe^{BP} is the cation fraction of Fe in the bulk planet (crust + mantle + core). Since total oxygen contents for PWDs are roughly Earth-like (Doyle et al. 2019), although perhaps slightly more oxidized on average, we apply a terrestrial $\alpha_{Fe} = 0.27$ (see Putirka and Rarick 2019). Finally, we test the algebraic methods of Hollands et all (2018) to calculate crust fractions of PWDs, using a mantle composition from McDonough and Sun (1995), an average crust composition from Rudnick and Gao (2014), an oceanic crust composition from Gale et al. (2013), and a core that contains 7% Si (Wade and Wood 2005) in addition to Fe. Hollands et al. (2018) use matrices to recast reservoir (core, mantle and crust) compositions into the mass fractions of each. In such a method, FeO in a silicate mantle is fixed, so fO_2 will implicitly vary for each PWD (using a fixed value for α_{Fe} a constant fO_2 is implicit and FeO contents in the mantle will vary; Table A2).

Bulk planetary compositions for the inner planets are computed, using metal core/silicate fractions of Szurgot (2015) and assuming that metal cores are 100 wt. % Fe, except for Earth, where we assume the core has 88 wt. % Fe and 7% Si (e.g., Wade and Wood 2005; Wood et al. 2014). We also compare PWDs to mean continental crust composition from Rudnick and Gao (2014), average mid-ocean ridge basalt from Gale et al. (2013), and to various rock types, including mid-ocean ridge and ocean island basalts, oceanic island arcs and picrites from various tectonic settings, from the GEOROC database

(http://georoc.mpch-mainz.gwdg.de/georoc/) and plutonic rocks of all types from the Sierra Nevada, as an example of upper continental crust, from the NAVDAT database (https://www.navdat.org/).

N.b.: the apparent positive and negative correlations in Fig. 1 in the main text are artifacts of the constant sum effect, which is amplified by projecting compositions into a four-component space that is dominated by three elements; see Chayes (1960) and Putirka and Rarick (2019). The positive correlation of Mg v Si for the terrestrial planets, for example, disappears ($R^2 = 0.19$) for MgO v. SiO_2 within a 10-oxide system.

Geochemical Projections

To test Hollands et al.'s (2018) identification and quantification of silicate crust fractions from extrasolar systems, we apply their algebraic approach to calculate crust, mantle and core fractions of the PWDs of Table 1. Their strategy was to use Earth-like compositions (using Ca, Mg and Fe) for the crust, mantle and core as inputs, so that for a three component system, a 3 x 3 matrix can relate bulk PWD compositions to the fractions of crust, mantle and core. Expressing the mass balance using row matrices, we have:

$$\left[C_{SiO_2}^{BPWD} \quad C_{FeO}^{BPWD} \quad C_{MgO}^{BPWD}\right] = \left[F_{Crust} \quad F_{Mantle} \quad F_{Core}\right] \begin{bmatrix} C_{SiO_2}^{Crust} & C_{FeO}^{Crust} & C_{MgO}^{Crust} \\ C_{SiO_2}^{Mantle} & C_{FeO}^{Mantle} & C_{MgO}^{Mantle} \\ C_{SiO_2}^{Core} & C_{FeO}^{Core} & C_{MgO}^{Core} \end{bmatrix} \qquad (A1.1)$$

or alternatively, as column matrices (where superscript T represents the transpose of the square matrix):

$$\begin{bmatrix} C_{SiO_2}^{BPWD} \\ C_{FeO}^{BPWD} \\ C_{MgO}^{BPWD} \end{bmatrix} = \begin{bmatrix} C_{SiO_2}^{Crust} & C_{FeO}^{Crust} & C_{MgO}^{Crust} \\ C_{SiO_2}^{Mantle} & C_{FeO}^{Mantle} & C_{MgO}^{Mantle} \\ C_{SiO_2}^{Core} & C_{FeO}^{Core} & C_{MgO}^{Core} \end{bmatrix}^T \times \begin{bmatrix} F_{Crust} \\ F_{Mantle} \\ F_{Core} \end{bmatrix} \qquad (A1.2)$$

where C_i^j is the weight % concentration of oxide (or element) i in reservoir j, F_j is the mass fraction of reservoir j and the superscript BPWD is the bulk composition of polluted white dwarfs (keeping in mind that renormalization is required after matrix multiplication). The Hollands et al. (2018) projection is in the system Ca-Mg-Fe, and as an independent, and perhaps more precise test (reported uncertainties on Si are much less than on Ca), we use the system Si-Fe-Mg. For our system, rearrangement of Equation A1 (where the superscript -1 indicates the inverse of a matrix) yields:

$$\left[F_{Crust} \quad F_{Mantle} \quad F_{Core}\right] = \left[C_{SiO_2}^{BPWD} \quad C_{FeO}^{BPWD} \quad C_{MgO}^{BPWD}\right] \times \begin{bmatrix} C_{SiO_2}^{Crust} & C_{FeO}^{Crust} & C_{MgO}^{Crust} \\ C_{SiO_2}^{Mantle} & C_{FeO}^{Mantle} & C_{MgO}^{Mantle} \\ C_{SiO_2}^{Core} & C_{FeO}^{Core} & C_{MgO}^{Core} \end{bmatrix}^{-1} \qquad (A2.1)$$

or alternatively, as column matrices (where the superscript T represents the transpose of a matrix):

$$\begin{bmatrix} F_{Crust} \\ F_{Mantle} \\ F_{Core} \end{bmatrix} = \left[\begin{bmatrix} C_{SiO_2}^{Crust} & C_{FeO}^{Crust} & C_{MgO}^{Crust} \\ C_{SiO_2}^{Mantle} & C_{FeO}^{Mantle} & C_{MgO}^{Mantle} \\ C_{SiO_2}^{Core} & C_{FeO}^{Core} & C_{MgO}^{Core} \end{bmatrix}^T\right]^{-1} \times \begin{bmatrix} C_{SiO_2}^{BPWD} \\ C_{FeO}^{BPWD} \\ C_{MgO}^{BPWD} \end{bmatrix} \qquad (A2.2)$$

.

For Eqn. A1, we use the bulk silicate Earth composition from McDonough and Sun (1995), a metallic core composition that contains 7 wt. % Si and 88 wt. % Fe (Wood et al. 2014) and we calculate Earth's bulk crust composition using the average continental crust of Rudnick and Gao (2014), the average oceanic crust composition of Gale et al. (2013), and crust masses from Petersen and DePaolo (2007), where the continental crust mass is 2.17 x 10^{22} kg, and the oceanic crust mass is 5.99 x 10^{21} kg. (Note: estimates of "bulk silicate Earth" are effectively equal to Earth's mantle. The mantle has a mass of ca. 4.0 x 10^{24} kg, and so the crust is just under 0.7 wt. % of the total mass of the silicate Earth and is inconsequential for the total abundances of the major elements or oxides). After renormalization so that SiO_2 + FeO + MgO + CaO = 100 for each reservoir, we have:

$$\begin{bmatrix} C_{SiO_2}^{Crust} & C_{FeO}^{Crust} & C_{MgO}^{Crust} \\ C_{SiO_2}^{Mantle} & C_{FeO}^{Mantle} & C_{MgO}^{Mantle} \\ C_{SiO_2}^{Core} & C_{FeO}^{Core} & C_{MgO}^{Core} \end{bmatrix} = \begin{bmatrix} 74.2 & 9.5 & 6.8 \\ 47.7 & 8.5 & 40 \\ 11.6 & 88.4 & 0 \end{bmatrix} \quad \text{(A2.3)}$$

Mineral modes (Figure 3 of main text) are calculated similarly, from bulk silicate planet (BSP) compositions (Table A2) expressed as mole fractions (X_i^{BSP}, where the subscript i is an oxide of interest, such as SiO_2, MgO, FeO or CaO), and the mole fractions of various mineral phases (X_i^j, where j is the mineral phase in question). This is effectively the method of Thompson (1982). The mineral fractions (F_i) of about half of PWDs can be described as positive (or negative but very nearly zero) amounts of olivine (Ol), clinopyroxene (Cpx), orthopyroxene (Opx) and wüstite (Wus); the mass balance expressed as row matrices is:

$$[F_{Ol} \; F_{Cpx} \; F_{Opx} \; F_{Wus}] = [X_{SiO_2}^{BSP} \; X_{FeO}^{BSP} \; X_{MgO}^{BSP} \; X_{CaO}^{BSP}] \times \begin{bmatrix} X_{SiO_2}^{Ol} & X_{FeO}^{Ol} & X_{MgO}^{Ol} & X_{CaO}^{Ol} \\ X_{SiO_2}^{Cpx} & X_{FeO}^{Cpx} & X_{MgO}^{Cpx} & X_{CaO}^{Cpx} \\ X_{SiO_2}^{Opx} & X_{FeO}^{Opx} & X_{MgO}^{Opx} & X_{CaO}^{Opx} \\ X_{SiO_2}^{Wus} & X_{FeO}^{Wus} & X_{MgO}^{Wus} & X_{CaO}^{Wus} \end{bmatrix}^{-1} \quad \text{(A3.1)}$$

or as column matrices, is:

$$\begin{bmatrix} F_{Ol} \\ F_{Cpx} \\ F_{Opx} \\ F_{Wus} \end{bmatrix} = \begin{bmatrix} \begin{bmatrix} X_{SiO_2}^{Ol} & X_{FeO}^{Ol} & X_{MgO}^{Ol} & X_{CaO}^{Ol} \\ X_{SiO_2}^{Cpx} & X_{FeO}^{Cpx} & X_{MgO}^{Cpx} & X_{CaO}^{Cpx} \\ X_{SiO_2}^{Opx} & X_{FeO}^{Opx} & X_{MgO}^{Opx} & X_{CaO}^{Opx} \\ X_{SiO_2}^{Wus} & X_{FeO}^{Wus} & X_{MgO}^{Wus} & X_{CaO}^{Wus} \end{bmatrix}^{T} \end{bmatrix}^{-1} \times \begin{bmatrix} X_{SiO_2}^{BSP} \\ X_{FeO}^{BSP} \\ X_{MgO}^{BSP} \\ X_{CaO}^{BSP} \end{bmatrix} \quad \text{(A3.1)}$$

where the supserscript -1 represents the inverse of a matrix and the superscript T represents the transpose of a matrix. To describe the mineral compositions in our "standard mineralogy", we use:

$$\begin{bmatrix} X_{SiO_2}^{Ol} & X_{FeO}^{Ol} & X_{MgO}^{Ol} & X_{CaO}^{Ol} \\ X_{SiO_2}^{Cpx} & X_{FeO}^{Cpx} & X_{MgO}^{Cpx} & X_{CaO}^{Cpx} \\ X_{SiO_2}^{Opx} & X_{FeO}^{Opx} & X_{MgO}^{Opx} & X_{CaO}^{Opx} \\ X_{SiO_2}^{Wus} & X_{FeO}^{Wus} & X_{MgO}^{Wus} & X_{CaO}^{Wus} \end{bmatrix} = \begin{bmatrix} 1 & 0.2 & 1.8 & 0 \\ 2 & 0.2 & 0.8 & 1 \\ 2 & 0.2 & 1.8 & 0 \\ 0 & 1 & 0 & 0 \end{bmatrix} \qquad (A4)$$

Which represents the specific mineral formulas as follows: olivine = $Mg_{1.8}Fe_{0.2}SiO_4$; clinopyroxene = $CaMg_{0.8}Fe_{0.2}Si_2O_6$; orthopyroxene = $Mg_{1.8}Fe_{0.2}Si_2O_6$; wüstite = FeO, which are typical compositions for mantle equilibrated conditions that are close to 1300°C and 2 GPa, which represents are "standard" of equilibration.

Those PWDs that have large negative amounts of olivine when applying Eqns. A3–A4 can be described as positive combinations of Wus, Cpx, Quartz (Qtz) and Opx, using the equations:

$$\begin{bmatrix} F_{Wus} \\ F_{Cpx} \\ F_{Qtz} \\ F_{Opx} \end{bmatrix} = \left[\begin{bmatrix} X_{SiO_2}^{Wus} & X_{FeO}^{Wus} & X_{MgO}^{Wus} & X_{CaO}^{Wus} \\ X_{SiO_2}^{Cpx} & X_{FeO}^{Cpx} & X_{MgO}^{Cpx} & X_{CaO}^{Cpx} \\ X_{SiO_2}^{Qtz} & X_{FeO}^{Qtz} & X_{MgO}^{Qtz} & X_{CaO}^{Qtz} \\ X_{SiO_2}^{Opx} & X_{FeO}^{Opx} & X_{MgO}^{Opx} & X_{CaO}^{Opx} \end{bmatrix}^{T} \right]^{-1} \times \begin{bmatrix} X_{SiO_2}^{BSP} \\ X_{FeO}^{BSP} \\ X_{MgO}^{BSP} \\ X_{CaO}^{BSP} \end{bmatrix} \qquad (A5)$$

using the following matrix:

$$\begin{bmatrix} X_{SiO_2}^{Wus} & X_{FeO}^{Wus} & X_{MgO}^{Wus} & X_{CaO}^{Wus} \\ X_{SiO_2}^{Cpx} & X_{FeO}^{Cpx} & X_{MgO}^{Cpx} & X_{CaO}^{Cpx} \\ X_{SiO_2}^{Qtz} & X_{FeO}^{Qtz} & X_{MgO}^{Qtz} & X_{CaO}^{Qtz} \\ X_{SiO_2}^{Opx} & X_{FeO}^{Opx} & X_{MgO}^{Opx} & X_{CaO}^{Opx} \end{bmatrix} = \begin{bmatrix} 0 & 1 & 0 & 0 \\ 2 & 0.2 & 0.8 & 1 \\ 1 & 0 & 0 & 0 \\ 2 & 0.2 & 1.8 & 0 \end{bmatrix} \qquad (A6).$$

Here we use the same mineral compositions of Eqn. A4, except that quartz (SiO_2) substitutes for olivine. Of course, one need not take the transpose of the transformation matrix A6 if multiplication involves row matrices, and the order of the matrix multiplication is conducted as in Eqn. A3.1.

Finally, those PWDs that have large negative amounts of Opx when applying Eqns. A3-A4 can be described as positive combinations of garnet (Gar), Cpx, Periclase (Per), and Ol, using the equations:

$$\begin{bmatrix} F_{Gar} \\ F_{Cpx} \\ F_{Per} \\ F_{Ol} \end{bmatrix} = \left[\begin{bmatrix} X_{SiO_2}^{Gar} & X_{FeO}^{Gar} & X_{MgO}^{Gar} & X_{CaO}^{Gar} \\ X_{SiO_2}^{Cpx} & X_{FeO}^{Cpx} & X_{MgO}^{Cpx} & X_{CaO}^{Cpx} \\ X_{SiO_2}^{Per} & X_{FeO}^{Per} & X_{MgO}^{Per} & X_{CaO}^{Per} \\ X_{SiO_2}^{Ol} & X_{FeO}^{Ol} & X_{MgO}^{Ol} & X_{CaO}^{Ol} \end{bmatrix}^{T} \right]^{-1} \times \begin{bmatrix} X_{SiO_2}^{BSP} \\ X_{FeO}^{BSP} \\ X_{MgO}^{BSP} \\ X_{CaO}^{BSP} \end{bmatrix} \qquad (A7)$$

and

$$\begin{bmatrix} X_{SiO_2}^{Gar} & X_{FeO}^{Gar} & X_{MgO}^{Gar} & X_{CaO}^{Gar} \\ X_{SiO_2}^{Cpx} & X_{FeO}^{Cpx} & X_{MgO}^{Cpx} & X_{CaO}^{Cpx} \\ X_{SiO_2}^{Per} & X_{FeO}^{Per} & X_{MgO}^{Per} & X_{CaO}^{Per} \\ X_{SiO_2}^{Ol} & X_{FeO}^{Ol} & X_{MgO}^{Ol} & X_{CaO}^{Ol} \end{bmatrix} = \begin{bmatrix} 4 & 0 & 4 & 0 \\ 2 & 0.2 & 0.8 & 1 \\ 0 & 0 & 1 & 0 \\ 1 & 0.2 & 1.8 & 0 \end{bmatrix} \quad \text{(A8)}$$

where the mineral compositions of Ol and Cpx are as in Eqn. A4, but we now add the following mineral compositions: periclase (MgO) and pyrope garnet ($Mg_4Al_3Si_4O_{12}$), the latter of which is compositionally indistinguishable from enstatite ($Mg_2Si_2O_6$) or majorite garnet, both of which have the composition $Mg_4Si_4O_{12}$ when expressed on the basis of 12 oxygens.

Extended Data

Table A1. Polluted white dwarfs: compositions as weight % cations, and stellar properties.

White Dwarf	Source	Si	Fe	Mg	Ca	Disk?[a]	Atm[b]	SpT[c]	T_{eff}(K)[d]	log(g)[e]	*D* (pc)[f]
PG 0843+517	Xu et al. (2019)	7.9	87.9	4	0.2	Y	H	DAZ	24,670	7.9	136
WD 1929+011	Melis et al. (2011)	16.3	57.8	25.1	0.8	Y	H	DAZ	23,470	8.0	53
WD 1536+520	Farihi et al. (2016)	24.7	32.5	38.9	3.9	Y	He	DBAZ	20,800	8.0	205
PG 1015+161	Xu et al. (2019)	12.5	78.5	7.1	1.9	Y	H	DAZ	20,420	8.1	87
Ton 345	Jura et al. (2015)	32.1	44.2	21.6	2.2	Y	He	DBZ	18,700	8.0	107
WD 1041+092	Melis and Dufour (2017)	32.6	9.8	46.8	10.9	Y	He	DBZ	18,330	8.1	174
HE 0106-3253	Xu et al. (2019)	7	84.6	4.9	3.5	Y	H	DAZ	17,350	8.1	69
GD 61	Farihi et al. (2013	38.2	12.6	44.6	4.5	Y	He	DBAZ	17,280	8.2	54
G 241-6	Jura et al. (2012)	22	27.7	43.7	6.6	N	He	DBZ	15,300	8.0	73
GD 40	Jura et al. (2012)	20.6	38.2	31	10.2	Y	He	DBZ	15,300	8.0	64
WD 1551+175	Xu et al. (2019)	31.9	33.3	26.4	8.5	Y	He	DBZ	14,756	8.0	159
WD 2207+121	Xu et al. (2019)	29.8	37.4	29.6	3.2	Y	He	DBZ	14,752	8.0	166
WD 1145+017	Fortin-Archambault et al. (2020)	17.5	66.2	14.4	1.9	Y	He	DBAZ	14,500	8.1	142
WD 1425+540	Xu et al. (2017)	31.6	48.2	17.9	2.2	N	He	DBAZ	14,490	8.0	52
HS 2253+8023	Klein et al. (2011)	19.8	49.5	25.3	5.4	N	He	DBZ	14,400	8.4	71
WD J0738+1835	Dufour et al. (2012)	24.1	39.8	34.6	1.6	Y	He	DBZ	13,950	8.4	173
WD J1242+5226	Raddi et al. (2015)	39.5	19.7	37.5	3.3	N	He	DBZ	13,000	8.0	161
G 29-38	Xu et al. (2014)	36.6	36.5	21.4	5.5	Y	H	DAZ	11,820	8.4	18
WD 1232+563	Xu et al. (2019)	23.3	37.6	37.5	1.6	?	He	DBZ	11,787	8.3	174
PG 1225-079	Xu et al. (2013)	23.5	50.1	18.1	8.2	Y	He	DZ	10,800	8.0	33
GD 362	Xu et al. (2013)	19	58.4	11.9	10.8	Y	He	DBZ	10,540	8.2	56
Ross 640	Koester and Wolff (2000)	33.8	10.7	52.1	3.4	N	He	DZ	8,500	8.0	16
NLTT 43806	Zuckerman et al. (2011)	34.8	17.4	37.9	9.9	N	H	DAZ	5,900	8.0	71
Sun[g]	Lodders and Fegley (2018)	*27.3*	*46.1*	*24.1*	*2.5*				*5,780*	*4.4*	

(a) Indicates whether a disk of dust/gas has been detected. (b) The dominant element in the stellar atmosphere. (c) Stellar type: DA = H atmosphere; DB = He atmosphere; Z = polluted by "metals" (e.g., atomic number >2); DBAZ = both H and He lines are detected in the stellar atmosphere. (d) T_{eff} is the "effective temperature" which is a blackbody temperature that approximates the temperature of a star's photosphere. (e) log of gravitational acceleration (cm/s^2). (f) Distance from Sun (Brown et al. 2018). (g) Solar composition.

Table A2. Polluted white dwarfs as wt. % oxides, and Bulk Silicate Planet (BSP) compositions

	Bulk Composition				Bulk Silicate Planet[a]				*Hollands et al.*	*This Study*
	SiO_2	FeOt	MgO	CaO	SiO_2	FeOt	MgO	CaO	$(F_{cr})(F_m)(F_{cst})$ [b]	$(F_{cr})(F_m)(F_{cst}))$ [c]
PG 0843+517	12.3	82.6	4.9	0.2	58.7	16.9	23.2	1.2		(0.87)(0.13)(0)
WD 1929+011	23.0	48.9	27.4	0.7	39.8	11.4	47.5	1.2		(0.41)(0.59)(0)
WD 1536+520	32.1	25.4	39.2	3.3	40.3	6.4	49.2	4.1	(0.23) (0.77) (0)	(0.17)(0.83)(0)
PG 1015+161	18.8	71.0	8.3	1.8	55.0	15.3	24.4	5.4	(0.366)(0.325)(0.309)	(0.79)(0.21)(0)
Ton 345	41.8	34.6	21.8	1.9	58.6	8.3	30.5	2.6		(0.32)(0.50)(0.18)
WD 1041+092	39.8	7.2	44.3	8.7	42.1	1.9	46.8	9.2		(0)(1.0)(0)
HE 0106-3253	10.9	79.5	6.0	3.6	44.1	17.3	24.1	14.5		(0.84)(0.16)(0)
GD 61	45.9	9.1	41.5	3.6	49.2	2.4	44.6	3.8	(0.032)(0.584)(0.384)	(0.01)(0.99)(0)
G 241-6	28.7	21.6	44.1	5.6	34.6	5.5	53.2	6.7	(0.096)(0.384)(0.52)	(0.13)(0.87)(0)
G D40	27.7	31.0	32.3	9.0	37.1	7.7	43.2	12.0	(0.098)(0.132) (0.77)	(0.26)(0.74)(0)
WD 1551+175	41.0	25.7	26.2	7.1	51.6	6.4	33.0	9.0		(0.23)(0.66)(0.11)
WD 2207+121	38.5	29.1	29.7	2.7	50.4	7.2	38.8	3.6		(0.26)(0.74)(0)
WD 1145+017	25.1	57.1	16.0	1.8	50.9	12.9	32.6	3.7	(0.457)(0.355)(0.188)	(0.60)(0.40)(0)
WD 1425+540	41.6	38.2	18.3	1.9	61.2	9.0	26.9	2.8		(0.36)(0.41)(0.23)
HS 2253+8023	27.2	41.0	27.0	4.8	41.5	9.8	41.2	7.4	(0.299)(0.327)(0.374)	(0.37)(0.63)(0)
WD J0738+1835	31.7	31.6	35.3	1.4	42.8	7.7	47.6	1.9		(0.24)(0.76(0)
WD J1242+5226	47.8	14.4	35.2	2.6	53.8	3.7	39.6	3.0	(0.123)(0.57) (0.307)	(0.07)(0.85)(0.08)
G 29-38	46.5	27.9	21.1	4.5	60.1	6.8	27.2	5.9	(0.175)(0.376)(0.448)	(0.24)(0.48)(0.28)
WD 1232+563	30.6	29.8	38.3	1.3	40.4	7.4	50.5	1.8		(0.21)(0.79)(0)
PG 1225-079	32.2	41.2	19.2	7.4	49.3	9.8	29.5	11.3	(0.19)(0.047) (0.763)	(0.44)(0.49)(0.07)
GD 362	27.0	49.9	13.1	10.0	47.5	11.7	23.1	17.7	(0.168)(0)(0.832)	(0.57)(0.34)(0.09)
Ross 640	40.8	7.7	48.7	2.7	43.4	2.0	51.7	2.9		(0)(1.0)(0)
NLTT 43806	42.9	12.9	36.2	8.0	47.6	3.3	40.2	8.9	(0.042)(0.38)(0.579)	(0.06)(0.94)(0)
Sun[d]	*36.2*	*36.8*	*24.8*	*2.1*	*52.2*	*8.9*	*35.8*	*3.1*		*(0.35)(0.61)(0.04)*

(a) Bulk silicate planet (BSP) compositions are calculated assuming that during core formation, Fe partitioning as on Earth, where α_{Fe} = Fe^{m+cst}/Fe^{BP} (Fe^{m+cst} = total Fe in the mantle and crust; Fe^{BP} is Fe in the bulk planet, i.e., core + mantle crust) and α_{Fe} = 0.27 (see Putirka and Rarick 2019). (b) Fractions of crust (F_{cst}), mantle (F_m) and core (F_{cr}) as calculated by Hollands et al. (2018), with renormalization so that their negative estimates become zero. (c) Fractions as in (b), as calculated in this study, using α_{Fe} = 0.27 and SiO_2, MgO and FeO for mass balance and continental crust from Rudnick and Gao (2014) and a mantle composition from McDonough and Sun (1995), renormalized so that SiO_2 + FeOt + MgO + CaO = 100%. (d) Solar composition (Lodders and Fegley 2018).

Table A3. Possible matches of PWDs with Solar System Rock Types

PWD	Meteorite/Lunar Types From Bulk PWD[a]	Terrestrial Rock Types From Bulk PWD[a]	Rock Types From PWD BSP[b]	Mantle Mineralogy From PWD BSP[c]
PG 0843+517	Iron	-	Shergottite	***Quartz Orthopyroxenite***
WD 1929+011	-	-	-	Dunite
WD 1536+520	-	-	-	***Periclase Dunite***
PG 1015+161	Mesosiderite	-	Shergottite	***Quartz Pyroxenite***
Ton 345	Shergottite	-	-	***Quartz Orthopyroxenite***
WD 1041+092	-	-	-	***Periclase Clinopyroxenite***
HE 0106-3253	Mesosiderite	-	Continental Picrite/Lunar Basalt	Wehrlite
GD 61	Achondrite	Continental Picrite	-	Lherzolite
G 241-6	-	-	-	***Periclase Wehrlite***
GD 40	-	-	-	***Periclase Wehrlite***
WD 1551+175	Shergottite/Lunar Basalt	Continental Picrite	Continental Picrite	Lherzolite
WD 2207+121	Chondrite/Shergottite	-	-	Lherzolite
WD 1145+017	-	-	-	Olivine Websterite
WD 1425+540	Shergottite	-	-	***Quartz Orthopyroxenite***
HS 2253+8023	-	-	-	***Periclase Wehrlite***
WD J0738+1835	-	-	-	Dunite
WD J1242+5226	Shergottite	Continental Picrite	-	Harzburgite or Aubrite
G 29-38	Shergottite/Lunar Basalt	-	-	***Quartz Pyroxenite***
WD 1232+563	-	-	-	***Periclase Dunite***
PG 1225-079	-	-	Continental Picrite/Lunar Basalt	Lherzolite
GD 362	-	-	Continental Picrite/Lunar Basalt	Olivine Clinopyroxenite
Ross 640	-	-	-	***Periclase Dunite***
NLTT 43806	Shergottite	Continental Picrite	-	Wehrlite
Sun	*Chondrite*	-	*Olivine Websterite*	*Olivine Websterite*

(a) Each rock type match is based on bulk PWD (polluted white dwarf) compositions (Fig. 1; Table A2) as compared to whole rock analyses of meteorites, Apollo mission samples and terrestrial rocks. (b) Rock type matches are as in (a), but for PWDs we use a “Bulk Silicate Planet” (BSP) composition, calculated by removing an Earth-like fraction of Fe to form a metallic core (Fig. 2; Table A2). (c) Mantle mineralogy calculated by mass balance using BSP compositions, and expressed as rock names using the ultramafic rock ternary of Le Bas and Streckeisen (1991; their Fig. 2; normal font in this table). All rock names in ***bold italic*** font are nominally exotic and, guided by Fig. 3, are named as follows: “quartz pyroxenites” have >10% each of orthopyroxene, clinopyroxene, and quartz; “quartz orthopyroxenites” have >10 % orthopyroxene and quartz, and < 10% clinopyroxene; “periclase dunites” have >10% each of periclase and olivine, and <10% clinopyroxene; “periclase wehrlites” contain >10% each of periclase, olivine and clinopyroxene; “periclase clinopyroxenites” have <10% olivine and >10% each of periclase and clinopyroxene.
